# Non-technical skills assessment scale in nursing: construction, development and validation^1^

**DOI:** 10.1590/1518-8345.2383.3042

**Published:** 2018-09-06

**Authors:** Sara Martins Pereira Pires, Sara Otília Marques Monteiro, Anabela Maria Sousa Pereira, Joana Novaes Machado Stocker, Daniela de Mascarenhas Chaló, Elsa Maria de Oliveira Pinho de Melo

**Affiliations:** 2 MSc, Researcher, Universidade de Aveiro, Departamento de Educação e Psicologia, Aveiro, Aveiro, Portugal. Researcher, Centro de Investigação em Tecnologias e Serviços de Saúde (CINTESIS), Aveiro, Portugal.; Centro de Investigação em Tecnologias e Serviços de Saúde, Aveiro, Portugal; 3 PhD, Assistant Professor, Universidade de Aveiro, Departamento de Educação e Psicologia, Aveiro, Aveiro, Portugal. Researcher, Centro de Investigação em Tecnologias e Serviços de Saúde (CINTESIS), Aveiro, Portugal.; Centro de Investigação em Tecnologias e Serviços de Saúde, Aveiro, Portugal; 4 PhD, Associate Professor, Universidade de Aveiro, Departamento de Educação e Psicologia, Aveiro, Aveiro, Portugal.; 5 MSc, Assistant Professor, Zayed University, College of Natural and Health Sciences, Dubai, Dubai, Emiratos Árabes Unidos.; 6 PhD, Assistant Professor, Universidade de Aveiro, Escola Superior de Saúde, Aveiro, Aveiro, Portugal.; 7 PhD, Adjunct professor, Universidade de Aveiro, Escola Superior de Saúde, Aveiro, Aveiro, Portugal.

**Keywords:** Non-Technical Skills, Crisis Resource Management, Healthcare, Nursing, Nursing Students, Psychometric Qualities

## Abstract

The introduction of non-technical skills during nursing education is crucial to prepare nurses for the clinical context and increase patient safety. We found no instrument developed for this purpose.

**Objectives:**

to construct, develop and validate a non-technical skills assessment scale in nursing.

**Method:**

methodological research. Based on the literature review and experience of researchers on non-technical skills in healthcare and the knowledge of the principles of crisis resource management, a list of 63 items with a five-point Likert scale was constructed. The scale was applied to 177 nursing undergraduate students. Descriptive statistics, correlations, internal consistency analysis and exploratory factor analysis were performed to evaluate the psychometric properties of the scale.

**Results:**

scale items presented similar values for mean and median. The maximum and the minimum values presented a good distribution amongst all response options. Most items presented a significant and positive relationship. Cronbach alpha presented a good value (0.94), and most correlations were significant and positive. Exploratory factor analysis using the Kaiser-Meyer-Olkin test showed a value of 0.849, and the Bartlett’s test showed adequate sphericity values (χ^2^=6483.998; p=0.000). One-factor model explained 26% of the total variance.

**Conclusion:**

non-technical skills training and its measurement could be included in undergraduate or postgraduate courses in healthcare professions, or even be used to ascertain needs and improvements in healthcare contexts.

## Introduction

The term non-technical skills (NTS) was primarily used in the aviation industry in a simulation-based training program for safety, known as crew resource management, designed to educate pilots and their crews about human performance limitations, understanding of cognitive errors, behavior analysis, communication, conflict-resolution and decision-making. The effective training prototype from aviation was adapted to healthcare contexts and became the crisis resource management (CRM), providing a simulation-based model for teaching NTS to healthcare professionals based on 15 acting principles: to know the environment, anticipate and plan, call for help early, exercise leadership and followership, distribute the workload, mobilize all available resources, communicate effectively, use all available information, prevent and manage fixation errors, cross (double) check, use cognitive aids, re-evaluate repeatedly, have a good teamwork, allocate attention wisely, and set priorities dynamically^1^.

The NTS training, such as in communication, teamwork, leadership, decision-making and situation-awareness, has proved to improve professionals’ performance^2^ and several healthcare courses and majors have recognized them as playing an important role to increase patients’ safety and achieve successful clinical outcomes. Indeed, it is now well acknowledged that NTS are essential skills to be acquired by different healthcare professionals^3^.

Specifically in undergraduate nursing courses, NTS training is the interface between the components of the real clinical context, in which future nurses will enter. Therefore, it is essential that nursing undergraduate students develop not only clinical and technical skills, but also NTS, since the challenges in the treatment of patients are often not due to lack of clinical expertise, but to failures in non-technical skills^2^. In order to effectively provide NTS training, it is essential to have an instrument to measure these skills. Several instruments have been developed to be used in various domains (operating room, resuscitation teams, obstetrics teams, trauma teams, trauma resuscitation, healthcare teams in acute settings and emergency environment), in order to meet this need^4^ ^-^ ^21^ in the context of specific multidisciplinary teams, working on a specific context, with specific procedures.

However, no theoretically based and easy-to-use assessment instrument has been published or developed and validated specifically for the assessment of NTS in the activities of nurses in general. Such an instrument is necessary to benchmark good NTS and to guide a formative feedback to the future practice of nursing students, and that is what we aim to discuss in this paper: to present the development and validation studies of a scale built based on theories and previous studies of NTS, specifically adapted for nursing undergraduate students, as it can be used to assess NTS in order to enable a greater understanding of these skills and enhance the performance of nursing undergraduate students in their future practice and patient safety^22^. 

In this sense, since there was no specific instrument for the context of nursing education, we carried out a panel discussion to adapt the CRM principles to the context of nursing practice, according to the language and the specific activities performed in nursing.

## Method

In order to develop the tool Non-Technical Skills - Nursing Assessment Scale (NTS-NAS), several phases were completed. Firstly, based on the literature review and the researchers’ experience on the topic, the research team constituted by nurses, nursing teachers, one anesthesiologist and three psychologists, developed a list of sentences (items) for each of the 15 key principles of CRM that would be the 15 dimensions of our scale (know the environment, anticipate and plan, call for help early, exercise leadership and followership, distribute the workload, mobilize all available resources, communicate effectively, use all available information, prevent and manage fixation errors, cross (double) check, use cognitive aids, re-evaluate repeatedly, have a good teamwork, allocate attention wisely, and set priorities dynamically). This process resulted in a list with 64 single-answer items with a five-point Likert scale, where students had to rate their level of agreement. Examples of items are: “I know every team member name”, “I call all patients by their names”. Based on the main topic, the assessment scale was entitled: “Non-technical skills assessment scale in nursing”. The scale was preceded by a set of instructions with the following content: “Given your scope of care, please complete the following questionnaire according to how you evaluate your usual performance. Use the scale of responses presented to evaluate each of the items. Choose the option “not applicable” when the item does not apply to your situation”. Secondly, all 64 items were reviewed by a panel discussion composed of three nursing experts and the study researchers who sought to identify possible gaps in the clarity of the statements, their representativeness for the construct and the content validity of each item, thus ensuring the validity of the construct. The panel discussed all items, one by one, until every member agreed that they were representative, observable, comprehensive and adequate to the competences of nursing undergraduate students. Furthermore, the experts also assessed the suitability of the items to the contexts of high- and low-fidelity clinical simulations. Some changes were made, such as: the panel discussion decided to eliminate the CRM principle/scale dimension “Mobilize all available resources”, due to its difficult measurement, the context and the fact that the nursing undergraduate students do not yet have autonomy to do so; some words have been replaced; some items were eliminated and other included; some items were removed from one principle and included in another one. Thirdly, the research team conducted a pre-test involving six senior nursing students to discuss and verify their understanding of NTS-NAS. Some changes in the instructions were necessary: “*Please, complete the following questionnaire according to how you evaluate your usual performance, taking into account your latest experience in a nursing team. Use the scale of responses presented to evaluate each of the items. Choose the option “Not applicable” when the item does not apply to your situation. It must be taken into account the definition of the following concepts: Scenarios: concerns the different diagnostic hypotheses/starting points, prior to decision making. Leader: concerns the person in charge of the care team*”.

The NTS-NAS and the informed consent forms were analyzed by the Director of the Nursing Course of the School of Health Sciences of the University of Aveiro and approved by the Scientific Committee of the Doctoral Program in Psychology of the University of Aveiro. Questionnaires were confidential, voluntary, anonymous, and collectively administered between October 2016 and January 2017, by the principal investigator to the nursing undergraduate students, in the classrooms, during regular school hours, and standardized oral instructions were given. Participants took between 5 and 15 minutes to answer. No major doubts emerged during the administration.

The central objective in the construction and development of the NTS-NAS was to evaluate the use of NTS in the nursing learning process, in order to be used in contexts of training in high- and low-fidelity clinical simulations. 

The NTS-NAS was constructed and developed in Portuguese, however, in this paper we will translate the necessary parts into English.

To select the sample, the following inclusion criteria were considered: there should be 2^nd^, 3^rd^ or 4^th^ grade nursing students, because clinical experience and knowledge were required to answer the scale; and exclusion criteria: 1^st^ grade nursing students (these undergraduate students have no clinical experience and knowledge yet to answer the scale).

The study version of the scale resulted in a list of 63 items, with a five-point Likert scale: “totally disagree”, “partially disagree”, “neither agree nor disagree”, “partially agree”, and “totally agree”, and the option “non-applicable”. It is subdivided into 14 dimensions that correspond to the 14 CRM principles: know the environment, anticipate and plan, call for help early, exercise leadership and followership, distribute the workload, communicate effectively, use all available information, prevent and manage fixation errors, cross (double) check, use cognitive aids, re-evaluate repeatedly, have a good teamwork, allocate attention wisely, and set priorities dynamically.

In order to analyze the psychometric properties of the NTS-NAS, SPSS (version 23.0) was used. The following statistical analyses were performed: descriptive statistics (for sensitivity); correlations; internal consistency (Cronbach’s alpha), and exploratory factor analysis. 

## Results 

The scale was applied to a random sample of 177 nursing undergraduate students from the School of Health Sciences of the University of Aveiro, Portugal.

Participants were of both genders (83.6% were female nursing undergraduate students and 16.4% were male nursing undergraduate students), distributed across the 2^nd^, 3^rd^ and 4^th^ grades (42.9%, 40.7%, and 16.4%, respectively), and all of them already had experience with clinical practice in their internships, but no experience in crisis resource management or in high-fidelity simulation.

Firstly, in the NTS-NAS with a 14 dimensions model, regarding the analysis of the sensitivity of the NTS-NAS, the use of descriptive statistics allowed the exploration of the measures of central tendency, dispersion and distribution (Table 1). 

**Table 1:** Measures of central tendency, dispersion, and distribution. Aveiro, Portugal, 2016

Dimension	Its*	Mean	Mode	Md^†^	SD^‡^	Min^§^	Max^||^	Skewness	Kurtosis
Know^¶^	8	33.07	37	34	3.83	18	40	-.78	.77
Antic**	8	32.54	38	33	3.95	19	40	-.37	-.05
Call^††^	5	23.53	25	25	2.03	16	27	-1.3	1.3
Exerc^‡‡^	11	47.82	55	48	5.95	28	61	-.58	.03
Distr^§§^	2	8.10	9	8	1.30	2	11	-.78	1.9
Comm^||||^	6	25.43	27	26	3.37	11	31	-.80	1.2
Infor^¶¶^	1	4.34	5	4	.71	2	5	-.80	.14
Prev***	1	4.32	4	4	.64	3	5	-.40	-.69
Cross^†††^	5	21.45	24	22	2.67	14	26	-.46	-.56
Use^‡‡‡^	2	8.11	8	8	1.51	4	12	-.28	-.33
Evalu^§§§^	4	17.13	16	17	2.06	12	21	-.23	-.76
Team^||||||^	7	29.53	30	29	3.37	21	38	.43	.42
Attent^¶¶¶^	2	9.03	10	9	1.06	6	10	-.62	-.77
Prior****	1	4.24	5	4	.80	2	6	-.60	-.43

*Its - Number of dimension items;†Md - Median;‡SD - Standard deviation;§Min - Minimum;||Max - Maximum;¶Know - Know the environment;**Antic - Anticipate and plan; ††Call - Call for help early;‡‡Exerc - Exercise leadership and followership;§§Distr - Distribute the workload;||||Comm - Communicate effectively;¶¶Infor - Use all available information;***Prev - Prevent and manage fixation errors;†††Cross - Cross (double) check;‡‡‡Use - Use cognitive aids;§§§Evalu - Re-evaluate repeatedly;||||||Team - Have a good teamwork;¶¶¶Attent - Allocate attention wisely;****Prior - Set priorities dynamically.

In general, the means of the dimensions of the NTS-NAS were not affected by extreme values (outliers). In turn, the skewness and kurtosis coefficients are close to the unit, which indicates nonexistent or minimal deviations of normality in terms of the distribution of participants. Finally, the maximum and minimum values are clearly distant from each other, which shows that the participants’ answers are generally well distributed amongst all response options. Therefore, it can be deduced from this that these indicators suggest that the subjects’ responses are within the parameters of the normal curve.

In general, all dimensions presented a significant and positive relationship, which suggests that the higher their NTS competency in one dimension, the higher it will also be in the other dimension, and vice-versa (Table 2).

The dimensions that most relate are “Know the environment” and “Exercise leadership and followership” (r=0.64); “Call for help early” and “Allocate attention wisely” (r=0.60); “Exercise leadership and followership” and “Distribute the workload” (r=0.60); and “Use all available information” and “Prevent and manage fixation errors” (r=0.62). In contrast, the dimensions that less relate are “Exercise leadership and followership” and “Use all available information” (r=0.21); “Prevent and manage fixation errors” and “Have a good teamwork” (r=0.19); and, “Use cognitive aids” and “Have a good teamwork” (r=0.22).

**Table 2:** Correlations between the dimensions of the non-technical skills assessment scale in nursing. Aveiro, Portugal, 2016

Dimension	2.	3.	4.	5.	6.	7.	8.	9.	10.	11.	12.	13.	14.
1.Know*	.57^†^	.33^†^	.64^†^	.57^†^	.46^†^	.32^†^	.35^†^	.36^†^	.40^†^	.41^†^	.24^†^	.44^†^	.38^†^
2.Antic^‡^		.40^†^	.53^†^	.50^†^	.44^†^	.42^†^	.48^†^	.34^†^	.49^†^	.54^†^	.31^†^	.43^†^	.35^†^
3.Call^§^			.41^†^	.38^†^	.42^†^	.35^†^	.36^†^	.44^†^	.24^†^	.53^†^	.32^†^	.60^†^	.39^†^
4.Exerc^||^				.60^†^	.47^†^	.21^†^	.28^†^	.40^†^	.34^†^	.31^†^	.29^†^	.45^†^	.40^†^
5.Distr^¶^					.56^†^	.27^†^	.35^†^	.42^†^	.35^†^	.42^†^	.39^†^	.48^†^	.44^†^
6.Comm**						.41^†^	.40^†^	.47^†^	.28^†^	.39^†^	.44^†^	.40^†^	.52^†^
7.Infor^††^							.62^†^	.47^†^	.37^†^	.38^†^	.28^†^	.26^†^	.41^†^
8.Prev^‡‡^								.38^†^	.36^†^	.40^†^	.19^§§^	.27^†^	.32^†^
9.Cross^||||^									.32^†^	.43^†^	.32^†^	.41^†^	.44^†^
10.Use^¶¶^										.43^†^	.22^†^	.30^†^	.27^†^
11.Evalu***											.30^†^	.50^†^	.33^†^
12.Team^†††^												.27^†^	.33^†^
13.Attent^‡‡‡^													.36^†^
14.Prior^§§§^													

*Know - Know the environment;†p<0.05 - Significance below 0.05;‡Antic - Anticipate and plan;§Call - Call for help early;||Exerc - Exercise leadership and followership;¶Distr - Distribute the workload;**Comm - Communicate effectively;††Infor - Use all available information;‡‡Prev -Prevent and manage fixation errors;§§p<0.01 - Significance below 0.01;||||Cross - Cross (double) check;¶¶Use - Use cognitive aids;***Evalu - Re-evaluate repeatedly;†††Team - Have a good teamwork;‡‡‡Attent - Allocate attention wisely;§§§Prior - Set priorities dynamically.

The analysis of Cronbach’s alpha revealed good internal consistency values for almost all 14 dimensions, with a critical value of 0.70 as reference (Table 3).

**Table 3:** Cronbach’s alpha values and corrected item-total correlation. Aveiro, Portugal, 2016

Dimension	Item	Alpha	Alpha if item deleted	Correlation
Know*	8	.77	Alpha always <	.39 - .60
Antic^†^	8	.73	Alpha > to .74 if item 12 excluded	.33 - .58
Call^‡^	5	.85	Alpha > to .87 if item 57 excluded	.50 - .68
Exerc^§^	11	.88	Alpha always <	.31 - .76
Distr^||^	2	.54		. 38
Comm^¶^	6	.74	Alpha always <	. 41- .60
Cross**	5	.68	Alpha always <	.34 - .61
Use^††^	2	.42		.27
Evalu^‡‡^	4	.71	Alpha always <	.39 - .62
Team^§§^	7	.36	Alpha > to .41 if item 50 excluded Alpha > to .55 if item 52 excluded	-.01-.36
Attent^||||^	2	.71		-.56

*Know - Know the environment;†Antic - Anticipate and plan;‡Call - Call for help early;§Exerc - Exercise leadership and followership;||Distr - Distribute the workload;¶Comm - Communicate effectively;**Cross - Cross (double) check;††Use - Use cognitive aids;‡‡Evalu - Re-evaluate repeatedly;§§Team - Have a good teamwork;||||Attent - Allocate attention wisely.

Indeed, most coefficients were above 0.70, with the exception of the dimensions “Cross (double) check” (0.68); “Distribute the workload” (0.54); “Use cognitive aids” (0.42); and “Have a good teamwork” (0.36). For the other dimensions, the coefficients were between 0.71 and 0.88, with the dimensions “Know the environment”, “Exercise leadership and followership” and “Call for help early” being the most consistent ones. These results suggest that the dimensions “Cross (double) check”, “Distribute the workload”, “Use cognitive aids”, and “Have a good teamwork” do not have a solid internal consistency and, hence, may not be assessing what they are supposed to assess. In addition, the dimensions “Use all available information”, “Prevent and manage fixation errors”, and “Set priorities dynamically” could not be assessed since they have only one item each. Considering the items in particular, the exclusion of four items could potentially benefit the internal consistency of the respective dimension. The corrected item-total correlation coefficients were also analyzed, which correspond to the correlation of each item with the total score of the respective dimension by excluding the item itself. Therefore, a low coefficient (bellow 0.30) suggests that the item does not measure the same construct measured by the other items included^23^. Overall, these correlations corroborate the results of internal consistency, since the dimension “Have a good teamwork” is the one that presents the lowest correlation coefficients, which means that probably some items are not fulfilling their role of measuring the dimension “Have a good teamwork” itself. Indeed, four items of this dimension present coefficients lower than 0.30: item 50 (-0.02); item 51 (0.27); item 52 (-0.01); and item 56 (0.28). Finally, items 44 and 45 are also pointed out here with a very low correlation with the general dimension “use cognitive aids” (0.27), which indicates that it may also not be measuring “use cognitive aids” itself.

**Figure 1:**
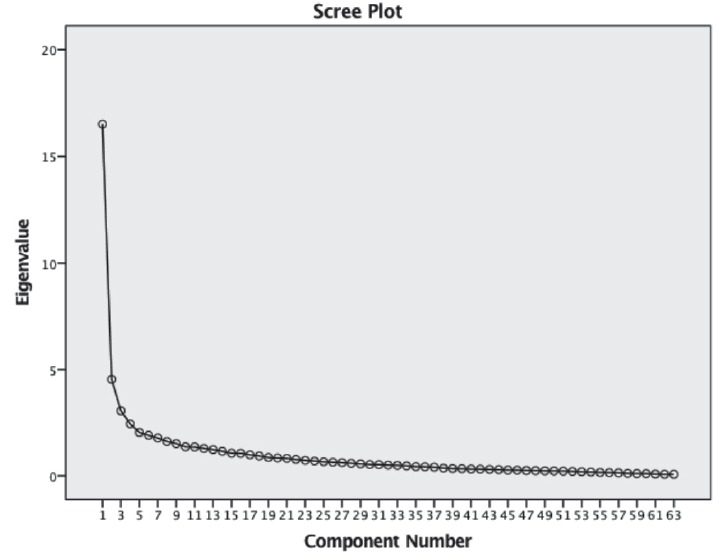
Scree Plot from the exploratory factor analysis of the non-technical skills assessment scale in nursing

Regarding the factorial validity or underlying structure of NTS-NAS, an exploratory factor analysis of principal components was performed using a varimax rotation and fixing 14 factors (corresponding to NTS-NAS dimensions). In the Kaiser-Meyer-Olkin (KMO) test, a value of 0,849 was obtained, which indicates a good adjustment of this factorial model to the present sample. In its turn, the Bartlett’s test also showed adequate sphericity values (χ^2^=6483.998; p=0.000), suggesting that the intercorrelation matrix differs from an identity matrix, and therefore, the variables of the NTS-NAS are correlated (as we had already confirmed). However, when analyzing the component matrix and the scree plot, there is a clear discrepancy between the first and the other 13 factors, as all 63 items are saturated in the first factor (Figure 1).

Therefore, we can assume that NTS may be better assessed in a unidimensional structure rather than in a multidimensional structure. Given these surprising and unexpected results of the factorial validity, a new assessment of the psychometric properties of the NTS-NAS was performed considering a unidimensional structure.

Sensitivity analysis was performed for all 63 items. In general, the items of the NTS-NAS present similar values for mean and median. Maximum and minimum values show that answers were well distributed amongst all response options. In addition, most skewness (skew) and kurtosis (kurt) coefficients are close to the unity, which indicates nonexistent or minimum deviations to normality in terms of participants’ distribution, except for the items: 4 (kurt= 1.475), 5 (kurt= 1.608), 19 (skew= -1.896; kurt= 3.480), 20 (kurt= 1.947), 24 (skew= -2.003; kurt= 8.315), 25 (skew= -2.606; kurt= 12.123), 30 (kurt= 3.047), 35 (kurt= 1.489), 51 (kurt= 1.633), 58 (skew= -1.586; kurt= 2.151), and 61 (skew= -2.251; kurt= 6.209).

Most items presented a significant and positive relationship, except for item 52 (“I got involved in conflict situations with other team members”) that presented a significant but negative correlation. This is because this is a negative item (it refers to the involvement in conflicts) while all the other items are formulated in a positive way. Therefore, a negative correlation between this item and the other items suggests that the higher their NTS competency, the less they get involved in conflict situations and vice versa. Items that relate the most are: 23-22 (r=0.83); 24-25 (r=0.73); 27-29 (r=0.69); 26-27 (r=0.68); 25-30 (r=0.67); 26-28 (r=0.65); 19-20 (r=0.63); 9-10 (r=0.62); and 15-16 (r=0.62). In contrast, the items that relate less are 1-40 (r=0.15); 9-35 (r=0.15); 15-23 (r=0.15); 26-47 (r=0.15); 28-49 (r=0.15); and 37-44 (r=0.15).

Some items presented a non-significant correlation, for example, items 1-11, 1-59, 2-19, 3-10 and 4-35. These results suggest that those items most related are referred to the same context or activities, and are integrated in the same CRM principle of action. And the contrary happens with those less or non-significantly related, although they also refer to NTS.

The analysis of Cronbach’s alpha revealed a good internal consistency value of 0.94. 

The corrected item-total correlation coefficients were also analyzed. Indeed, four items presented coefficients lower than 0.30: item 13 (0.29); item 40 (0.28); item 52 (-0.02); and item 53 (0.12). 

Regarding the factorial validity of NTS-NAS, an exploratory factor analysis of principal components was performed fixing one factor, as previously discussed. In the Kaiser-Meyer-Olkin (KMO) test, it was obtained the value of 0.849, which indicates a good adjustment of this factorial model to the present sample. Bartlett’s test also showed adequate sphericity values (χ^2^=6483.998; p=0.000), suggesting that the intercorrelation matrix differs from the identity matrix and, therefore, NTS-NAS variables are correlated (as we had already confirmed). The total model explained 26% of the total variance. In general, the factor loadings were between 0.37 and 0.73, which suggests that the items are influenced by the underlying factor and, therefore, belong to this unidimensional model. In addition, items presented commonality values between 0.24 and 0.53. 

## Discussion

Some of the results for the NTS-NAS with 14 dimensions were satisfactory, presenting good sensitivity, correlations and internal consistency, however, the exploratory factor analysis made it clear that a multidimensional structure with 14 dimensions is not viable. Surprisingly, this analysis pointed out the possibility of a NTS-NAS with an unidimensional structure. This may be because, in general, all items measure the same construct (NTS), and it may not be subdivided. Considering this unidimensional model, most of the results were also satisfactory, except for the skewness and kurtosis of some items, which may be due to the fact that the students did not want to compromise themselves in the disagreement options of the scale, answering what is expected of them to know and behave (social desirability). In another way, the reason why some items presented a non-significant correlation can be explained by the fact that although they integrate NTS, they do not have to do with each other in the sense that they refer to different contexts and activities (for example, item 2 “I know the equipment/clinical material that is available”, and item 19 “The team leader is clearly established”). Regarding the one-factor analysis of variance, the results were in general satisfactory, with the unidimensional model explaining 26% of the total variance.

To conclude, the NTS-NAS was built based on the 14 CRM principles and it was expected that 14 dimensions would be found, however, a unidimensional structure emerged for this questionnaire, which seems to be valid. In this sense, the final version of NTS-NAS resulted in a list of 63 items, with one dimension, NTS, and with a five-point Likert scale: “totally disagree”, “partially disagree”, “neither agree nor disagree”, “partially agree”, and “totally agree”, and a “non-applicable” option.

## Conclusion

This research was conducted in order to construct, develop and validate an instrument capable of measuring and representing NTS in nursing practice. This instrument seems to be appropriate to adequately assess NTS in nursing clinical contexts, however, more studies are needed to further validate the unidimensional model NTS-NAS, with a more representative sample of students/professionals from different healthcare settings. On the one hand, it is suggested that this instrument can be used in training settings, both in curricular internships and in specific workshops/intervention programs focused not only on technical habilities, but also on NTS. These types of intervention and respective assessment may significantly improve the performance, confidence, and self-efficacy of nursing students, and be an added value, as they can help them to better adjust to the complex clinical context, improve their clinical performance and ultimately, contribute to the safety and well-being of patients. On the other hand, NTS training and its measurement by using the NTS-NAS could also be included in postgraduate courses in healthcare professions and even be used to ascertain needs and improvements in healthcare contexts, such as in hospitals and private practices.
